# Exposure assessment of *Campylobacter* in United States broiler processing plants – Part 2: prevalence model

**DOI:** 10.1016/j.psj.2025.106011

**Published:** 2025-10-23

**Authors:** Rafael E. Rivera, Jinquan Wang, Abhinav Mishra, Harshavardhan Thippareddi, Sanjay Kumar, Manpreet Singh

**Affiliations:** a Department of Poultry Science, University of Georgia, Athens, GA 30602; b Department of Poultry Science, Auburn University, 260 Lem Morrison Dr, Auburn, AL, 36849; c Department of Food Science & Technology, University of Georgia, Athens, GA 30602; d Quality Technical Services and Food Safety, Niagara Bottling, Diamond Bar, CA 91765

**Keywords:** *Campylobacter*, QMRA, Poultry parts, Comminuted poultry, Interventions

## Abstract

Poultry processing plants implement controls to reduce *Campylobacter* prevalence and mitigate gastrointestinal disease risks. Quantitative microbial risk assessments (QMRA) use exposure assessments to evaluate *Campylobacter* changes and intervention efficacy in U.S. poultry processing plants. However, exposure assessments for poultry parts and comminuted products, representing higher share of total poultry consumption, have not been done till date. In this continuation of the exposure assessment, we conducted a systematic review and meta-analysis (SR-MA) to establish baseline for *Campylobacter* prevalence without interventions and assess intervention efficacy on carcasses, cut-up parts, and comminuted poultry. Initial prevalence (82 %) was calculated from literature. Odds ratios (OR) were calculated to indicate changes in *Campylobacter* prevalence. The scalding OR of 0.15 and chilling OR of 0.32 achieved the greatest reduction (*P* < 0.05). A baseline model showed prevalence reductions to 18.4 % for whole birds, 62.5 % for parts and 25.2 % for comminuted product. Validation against commercial integrator data (whole birds: <10 %; parts: ≤ 15 %) confirmed model robustness. A multi-hurdle intervention reduced prevalence in whole birds (2.14 %), cut-up parts (6.5 %), and comminuted (2.62 %). Interventions effectively reduce prevalence and variability across parts categories, improving exposure estimates.

## Introduction

*Campylobacter* is an important cause of zoonotic bacterial gastrointestinal infection in the United States, with symptoms including diarrhea, fever, stomach cramps, nausea, vomiting, and severe outcomes like death (CDC, 2022a). Annually, ∼ 20 cases per 100,000 people are diagnosed totaling 1.5 million infections (CDC, 2022b). *Campylobacter* is commonly associated with the consumption of poultry products (Berghaus et al., 2013; Ellis-Iversen et al., 2012; Overesch et al., 2020). In 2019, the Interagency Food Safety Analytics Collaboration (IFSAC) reported that over 80 % of non-dairy foodborne illnesses were attributed to chicken, other seafood (such as shellfish) and turkey, with *Campylobacter* illnesses most often linked to chicken (IFSAC, 2021).

The U.S. Department of Agriculture, Food Safety, and Inspection Service (USDA-FSIS) regulates safe poultry products through the Pathogen Reduction; Hazard Analysis and Critical Control Points rule (PR: HACCP Rule) in 1996 (USDA-FSIS, 1996). The USDA-FSIS has relied on controlling pathogens by developing performance standards aimed at reducing prevalence at the processing plant (USDA-FSIS, 1996). Since the introduction of the PR: HACCP rule, USDA-FSIS conducted several baseline surveys to determine *Salmonella* and *Campylobacter* prevalence on broiler carcasses in poultry processing establishments and revised the performance standards. The USDA-FSIS baseline survey, conducted in 1995, reported a *Campylobacter* prevalence of 88.2 % on chicken carcasses, which dropped to 18.3 % in 2019 (Williams et al., 2021), yet illness rates remain unchanged (CDC, 2022b). Recent data show persistent prevalence: carcasses (20.94 %, 2022), parts (16.75 %), and comminuted products (5.93 %) (USDA-FSIS, 2023).

The regulatory approach has targeted *Salmonella* and *Campylobacter* prevalence reduction at the processing plants through antimicrobial interventions. Poultry processors are constantly adjusting interventions to meet performance standards (Wideman et al., 2016). Antimicrobial interventions (e.g., chlorine, peroxyacetic acid (PAA)) are critical, though chlorine efficacy is pH- and organic matter dependent (Chen et al., 2020). Chlorine, typically used as sodium hypochlorite, has seen a decrease in use in favor of peroxyacetic acid PAA (DeVillena et al., 2022; Kataria et al., 2020). PAA provides a strong oxidizing function that disrupts the permeability of cell membranes and alters protein synthesis (Oyarzabal, 2005). PAA outperforms alternatives like cetylpyridinium chloride (CPC), trisodium phosphate (TSP), acidified sodium chlorite (ASC), and chlorine dioxide (ClO_2_) (Chen et al., 2014; Oyarzabal, 2005; Zhang et al., 2018).

Quantitative microbial risk assessments (QMRA) allow for risk-based evaluations for controlling microbial contamination and is becoming widely used to analyze food supply chains and their intervention strategies for microbial control (Ntakiyisumba et al., 2024). Several QMRAs that characterize *Campylobacter* contamination throughout the farm-to-fork continuum have been published. QMRA model comparison is ineffective as defined and consistent criteria are not used for development (Chapman et al., 2016). There is still a need to incorporate assessments from the different production components to characterize risk (Chapman et al., 2016). Exposure assessments are components within QMRAs that analyze the contribution of food processing to the spread of pathogens to the final consumer. Exposure assessments can be conducted using systematic reviews and meta-analysis to obtain baseline data to construct processing plant models to evaluate process improvements.

The systematic reviews utilize data from published experimental interventions from literature and can be used to develop models that identify where its use is most effective. It is important to observe the differences between poultry processing without interventions versus processing with interventions on all possible finished products to characterize which may present higher exposure risks to the consumer. Recent risk assessments and systematic reviews are limited to using whole carcass *Campylobacter* population data and do not include comparisons of poultry cut-up parts and comminuted poultry (Chapman et al., 2016; Dogan et al., 2022, 2019; Golden and Mishra, 2020; Keener et al., 2004; Sahin et al., 2015). Cut-up parts and comminuted poultry are the most consumed raw product in the U.S. and interventions must be evaluated and characterized to reduce the exposure risk (NCC, 2023). Comminuted product includes chicken that has been ground, chopped, shredded, or minced. An exposure assessment targeting *Campylobacter* populations in U.S. processing plants was performed confirming that common ready to cook poultry processing is capable of reducing *Campylobacter* concentrations using minimal interventions (Rivera et al., 2025). Therefore, an additional assessment on *Campylobacter* prevalence analysis in chicken cut-up parts and comminuted product in the U.S. should be collected and incorporated into future exposure assessments for better QMRAs and improved illness risk calculations.

The objective of this study is to estimate the *Campylobacter* prevalence in chicken parts and comminuted product that can potentially reach consumers through an exposure assessment. Prevalence will be obtained by 1) developing baseline *Campylobacter* prevalence in chicken cut-up parts and comminuted product in U.S. processing plants through a systematic review and meta-analysis and 2) estimating the efficacy of processing interventions in reducing *Campylobacter* prevalence in cut-up chicken parts, and comminuted product through simulation modeling. This assessment is a continuation of the work presented in Rivera et al. (2025).

## Materials and methods

### Systematic review and meta-analysis

**Fig. 1:**
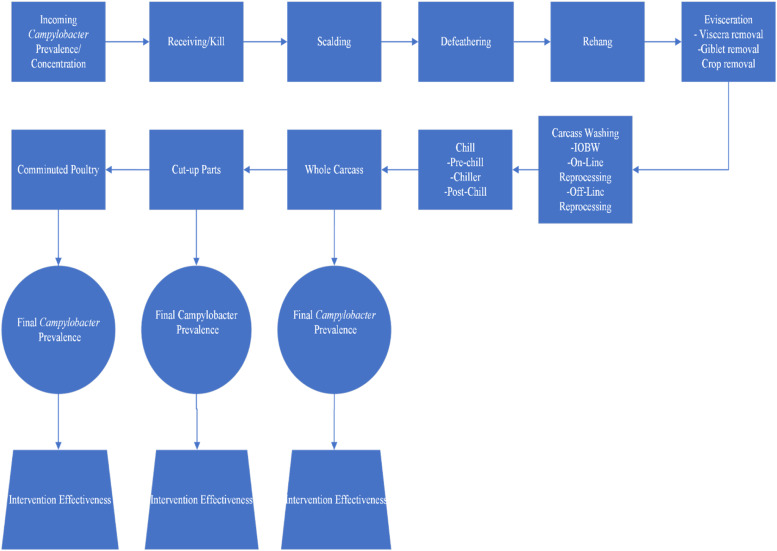
Flow diagram of poultry processing stages used for exposure assessment. Abbreviations: IOBW = inside-outside bird washer.

***Model Flow Chart.*** A flow chart (Fig. 1) was developed to model *Campylobacter* prevalence across poultry processing stages (receiving to grinding). Initial prevalence of *Campylobacter* at the receiving stage, without intervention or chlorine treatment, was defined as baseline or control. The chicken processing stages include scalding, feather picking, rehang, evisceration, carcass washing, immersion chilling, parts cut-up and grinding (comminuted) as standard operations of processing in the U.S. Final *Campylobacter* prevalence data in cut-up parts, and comminuted products were analyzed to evaluate process and/or intervention efficacy. Additionally, the changes in *Campylobacter* population in each subsequent processing stage up to the grinding stage were estimated. Changes in bacterial prevalence at each stage were quantified as an odds ratio (OR). Data for risk assessment inputs were obtained through a systematic review of literature and meta-analysis.

***Literature Search and Inclusion Criteria.*** A systematic review was adapted from Golden and Mishra (2020), and Sargeant and O'Connor (2014) to address the following research question:

1. How does *Campylobacter* prevalence on broiler carcasses change at each stage of processing from receiving to chicken parts and comminuted product in the U.S.?
2. What is the efficacy of chemical intervention and processing equipment on reducing *Campylobacter* prevalence and their interactions in the U.S.?

To address this question, the Web of Science (www.webofknowledge.com) and PubMed (https://pubmed.ncbi.nlm.nih.gov/) were searched using keywords aimed at addressing the research questions: (“*Campylobacter*” or “*Campylobacter jejuni*” or “*C. jejuni*”), AND (“United States” or “U.S.”) AND (“Poultry” or “Broiler” or “Chicken”) and “Intervention” and “Processing” and (“Prevalence” or “Isolation”) and data up to January 2023 was retrieved. In the absence of geographic description of the study, the location was inferred by the first and corresponding address. Additional studies were identified by searching review articles or other reference lists by hand. Inclusion criteria for accepting data includes, 1) Peer- reviewed, English-language primary research studies, excluding reviews; 2) Conducted in U.S. commercial/pilot plants; 3) Reported *Campylobacter* prevalence (%) in whole carcasses, parts, or comminuted products; 4) Tested interventions (chemical/equipment-based) with before-after or challenge study designs. All references were managed by the EndNote citation manager (Endnote 20, Clarivate Analytics, Philadelphia, PA). Duplicates were removed from EndNote by using the “find duplicates” function or manually.

Challenge studies were included only where commercial data was insufficient, with caveats regarding potential overestimation of intervention efficacy. Non-U.S. studies, review, and non-English articles were excluded.

***Inclusion Criteria.*** Abstracts were screened to determine eligibility with the following criteria included: 1) English language; 2) peer-reviewed journal articles; 3) primary research studies, excluding reviews; 4) interventions tested at a processing stage; 5) intervention tested on whole carcasses, cut-up parts, or ground chicken product. The prevalence from before-after studies in U.S. commercial broiler processing environments, and interventions tested in pilot plants needs to be reported for including in the meta-analysis. Commercial processing establishment before-after studies in different languages other than English or regions other than the U.S. were excluded from review. Following the initial screening, full-text articles were obtained for the remaining studies and analyzed for inclusion in model assessment. Studies with uncertain eligibility were reviewed and discussed by the authors until a consensus was reached.

Along with the previously mentioned screening criteria, details on the type of intervention used, application method, and necessary data to perform a meta-analysis (i.e., samples size, mean, standard deviation, confidence intervals, standard error of the mean, number of positive samples for prevalence) were evaluated as additional inclusion criteria.

***Data Extraction.*** Extracted data from literature included the number of initial and final *Campylobacter* positive samples and sample size, study type, processing step, type of intervention, intervention application method. The data were directly collected if the table is available, whereas the Plot Digitizer tool (Plot Digitizer, 3.1.5, 2024, https://plotdigitizer.com) was used to extract the prevalence values from the figures. Positives were further calculated from sample size.

***Quality Assessment of Included Studies.*** While systematic reviews and meta-analysis typically assess study quality, quality scoring was omitted here to avoid selection bias stemming from variability in scoring methodologies and their influence on meta-analytical interpretations (Stone et al., 2019).

***Data Analysis.*** All data analysis was performed using R version 4.0.1 (*R Core Team*, 2024). Meta-analyses and forest plot generation were conducted using the meta package (Schwarzer, 2007). A baseline model of *Campylobacter* prevalence at each stage of poultry processing establishments in the U.S. was constructed using a generalized linear mixed model. A logit link was used to stabilize the variance for the model. For each included study, prevalence values at each stage of broiler processing were calculated by dividing the sample size by the number of positive samples. The prevalence of *Campylobacter* was first transformed using the logit transformation:

$$\text{logit}\;p=\text{ln}\left(\frac{p}{1-p}\right)$$

with variance

$$\text{var}\;\left(\text{logit}\;p\right)=\frac{1}{Np}+\;\frac{1}{1-Np}$$

where p is the prevalence of *Campylobacter* reported in a study at a specific processing stage and N is the sample size of that study. A post-hoc comparison of all the processing stage were performed using multcomp (Hothorn et al., 2016) with Tukey multiple comparison correction.

For studies reporting changes in prevalence with a binary outcome, the number of positive samples and the total sample size were extracted from both the treatment and control groups. Odds ratios (OR) were calculated and used in the meta-analysis as the effect size based on the following formula:

$$OR=\frac{\left(\frac{p_{\text{treatment}}}{1-p_{\text{treatment}}}\right)}{\left(\frac{p_{\text{control}}}{1-p_{\text{control}}}\right)}$$

Where p_treatment_ and p_control_ is the prevalence in the treatment intervention and control group. An OR < 1 indicates a decrease in prevalence, an OR = 1 indicates no change, and an OR > 1 indicates an increase in prevalence (Dogan et al., 2022).

Inverse variance weighting was used to pool the prevalence studies. In the presence of zero-cell counts in either the treatment or control groups, a continuity correction of 0.5 was applied to all affected cells in the 2 × 2 table (Higgins et al., 2019; Sweeting et al., 2004).

Random-effects meta-analyses were performed with subgroup analyses of data based on the groups (i.e., interventions and intervention methods). The between-study variance (τ^2^) was estimated using the DerSimonian and Laird method (DerSimonian and Kacker, 2007; Schwarzer et al., 2015). The effect of heterogeneity was quantified on a relative scale using the *I*^2^ value with thresholds for interpretation as follows: 0 to 40 % heterogeneity might not be important, 30 to 60 % moderate heterogeneity, 50 to 90 % substantial heterogeneity, and 75 to 100 % considerable heterogeneity (Higgins et al., 2019, 2003; Schwarzer et al., 2015).

The estimated prevalence and OR in the forest plot is displayed along with a 95 % confidence interval (CI) along with the tau-squared (τ^2^) variance that describes the variance between the studies (Higgins et al., 2019) and I^2^ as the measure of heterogeneity. The value of I^2^ up to 40 % was considered low, 30–60 % was considered moderate, 50–90 % was considered substantial, and beyond 75 % was considered high (Deek et al., 2019).

***Publication Bias Assessment.*** Funnel plot asymmetry test for publication bias requires ≥10 studies and low heterogeneity (I^2^ < 50 %). As these criteria was not met, publication bias was not performed.

### Exposure assessment

***Processing Plant Module Overview***. The first objective of this module was to estimate *Campylobacter* prevalence (%) in whole birds, cut-up parts, and comminuted poultry under chlorine or no-interventions conditions, spanning stages from receiving to griding. The second objective was to estimate interventions efficacy in reducing *Campylobacter* during processing.

***Baseline Prevalence Estimate.*** Initial *Campylobacter* prevalence at the receiving stage were obtained from SR-MA results. The processing model incorporated standard U.S. processing stages (scalding, feather picking, rehang, evisceration, carcass washing, carcass chilling, parts cut up and grinding), with baseline inputs pooled from trials lacking reported interventions or reported chlorine use. Chlorine was used as part of the baseline since this intervention has historically been used for pathogenic bacterial control. It was assumed that commercial processing plant studies without reported use of interventions in control trials were using chlorine at the time of sampling.

OR values, modeled as pert distributions, were simulated using Monte Carlo simulation by Latin Hypercube Sampling with 10000 iterations using @Risk (version 8.4.1 (Build10), Palisade Company LLC, New York, USA). Baseline outputs informed for whole bird, chicken cut-up parts, and comminuted chicken were obtained for intervention efficacy analysis.

***Baseline Validation.*** Pre- and post-processing *Campylobacter* prevalence recovered from routine testing of 33 commercial processing facilities from two U.S. commercial chicken integrators over the period from 2018 to 2024. Samples with a limit of detection (LOD) of 1 CFU/mL included receiving, post-scalder, post-feather picking, rehang, post-chill, cut-up parts, and mechanically separated chicken (MSC) .Prevalence variations across parts (bone-in, boneless breast, tenders, fillets, wings, etc.) were analyzed using chi-square test (*P* < 0.05) in R (*R Core Team*, 2024). Quantification samples that were below the LOD were deemed negative.

***Intervention Efficacy Analysis.*** Single interventions (such as replacing immersion chilling with air chilling) or added steps (e.g., post-chill dips) obtained from the SR-MA were evaluated for its efficacy. The OR derived from the SR-MA were fit as a pert distribution where the 5th and 95th percentiles were the minimum and maximum value respectively and the observed mean OR its most likely value. Results from the interventions were expressed as 1. Mean *Campylobacter* prevalence (%) with its 95 % CI and 2. Intervention efficacy calculated using equation:

$$Intervention\;efficacy=\;\frac{Prevalence_{baseline}-\;Prevalence_{intervention}}{\;Prevalence_{baseline}}\;X\;100,$$

where $Prevalence_{baseline}$ refers to the mean *Campylobacter* prevalence for either whole birds, cut-up parts or comminuted chicken and $Prevalence_{intervention}$ refers to the mean *Campylobacter* prevalence for the alternative intervention scenario. Multiple intervention scenarios (pre- and post-chill) were also evaluated for cumulative effects on *Campylobacter* prevalence in whole birds, cut-up parts and comminuted chicken.

## Results

### Systematic review and meta-analysis

**Fig. 2:**
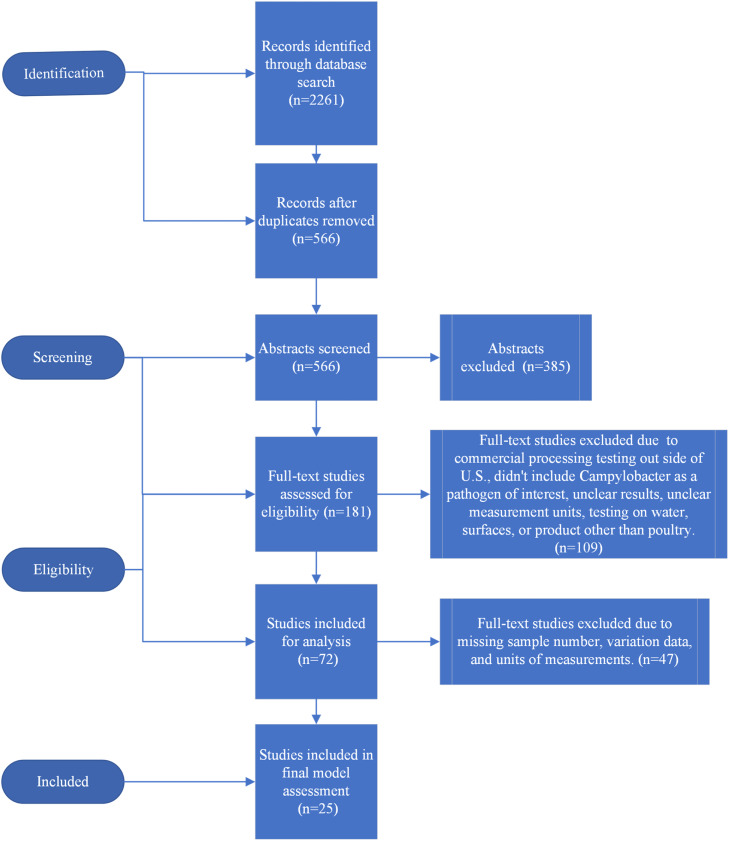
Flow chart of the systematic review process.

***Search Results.*** The initial search criteria produced 2,261 studies. After removing duplicates and screening the titles and abstracts, 181 records were retained for full text screening. A total of 72 records were retained for analysis after full text screening. 47 records were excluded due to missing sample number, variation data, and prevalence. A total of 21 commercial plants before/after studies, 2 pilot plants before/after studies, 2 years of USDA-FSIS performance standard samples for whole carcasses, parts, and comminuted chicken, totaling 25 studies were included for the meta-analysis and systematic review. The overview of the systematic review process is illustrated in Fig. 2.

***Characteristics of Included Studies.*** The characteristics of the included studies in the meta-analysis and risk assessment model are presented in Table 1. 9 studies reporting *Campylobacter* prevalence at receiving or prior to the scalding step were used to determine an incoming prevalence. 20 studies used chlorine or did not report an intervention in its control group. The control group consisted of trials without reported use of an intervention or chlorine use. Chlorine was used as part of the baseline since this intervention has historically been used for pathogenic bacterial control. It was assumed that commercial processing plant studies without reported use of interventions in control trials were using chlorine at the time of sampling. These were used to determine the baseline *Campylobacter* concentration and concentration change for each processing stage.

**Table 1:** Characteristics of included studies from systematic review.

Reference	Study Type	Equipment	Stage	Treatment	Sample Type	Rinsate	Enrichment Broth	Plating
Bailey et al. (2019)	Commercial plant before/after study		Receiving		Fecal/Ceca/Colon	PBS	Bolton Broth	Campy-Cefex
Berghaus et al. (2013)	Commercial plant before/after study		Receiving		Carcass Rinse	BPW	Bolton Broth	Campy-Cefex
Berrang and Dickens (2000)	Commercial plant before/after study		Receiving		Carcass Rinse	Distilled Water	PBS	Campy-Cefex
Berrang et al. (2011)	Commercial plant before/after study		Receiving		Carcass Rinse	Butterfield's Buffer	PBS	Campy-Cefex
DeVillena et al. (2022)	Commercial plant before/after study		Receiving		Carcass Rinse	BPW		Tempo
Kotula and Pandya (1995)	Commercial plant before/after study		Receiving		Carcass Rinse	Lactose Broth		Campy-BAP
Mead et al. (1995)	Commercial plant before/after study		Receiving		Neck Skin	MRD	Preston Broth	mCCDA
Potturi-Venkata et al. (2007)	Commercial plant before/after study		Receiving		Fecal/Ceca/Colon		Preston Broth	mCCDA/ Campy-Cefex
Son et al. (2007)	Commercial plant before/after study		Receiving		Carcass Rinse	Sterile Water	Bolton Broth	Campy-Cefex
Berrang and Dickens (2000)	Commercial plant before/after study	Scalder	Scalding	Chlorine	Carcass Rinse	Distilled Water	PBS	Campy-Cefex
Berrang et al. (2003)	Commercial plant before/after study	Scalder	Scalding	None	Carcass Rinse	PBS	PBS	Campy-Cefex
Berrang et al. (2011)	Commercial plant before/after study	Scalder	Scalding	None/High pH (Calcium Hydroxide)	Carcass Rinse	Butterfield's Buffer	PBS	Campy-Cefex
Berrang et al. (2019)	Commercial plant before/after study	Feather Picker	Post-Pick	None	Carcass Rinse	nBPW	Bolton Broth	Campy-Cefex
Berrang et al. (2001)	Pilot plant before/after study	Feather Picker	Post-Pick	None/Chlorine	Sponge	PBS		Campy-Cefex
Berrang et al. (2001)	Pilot plant before/after study	Feather Picker	Post-Pick	Cloacal Plug	Sponge	PBS		Campy-Cefex
Berrang and Dickens (2000)	Commercial plant before/after study	Feather Picker	Post-Pick	Chlorine	Carcass Rinse	Distilled Water	PBS	Campy-Cefex
Berrang et al. (2011)	Commercial plant before/after study	Dip Tank	Post-Pick	Chlorine	Carcass Rinse	Butterfield's Buffer	PBS	Campy-Cefex
Musgrove et al. (1997)	Commercial plant before/after study	Feather Picker	Post-Pick	None/Cloacal Plug	Carcass Rinse	PBS		Campy-Cefex
Thames et al. (2022)	Commercial plant before/after study	Feather Picker	Post-Pick	PAA	Carcass Rinse	BPW	Bolton Broth	Campy-Cefex
Berghaus et al. (2013)	Commercial plant before/after study	Rehang	Rehang	None	Carcass Rinse	BPW	Bolton Broth	Campy-Cefex
Berrang et al. (2007)	Commercial plant before/after study	Rehang	Rehang	Chlorine	Carcass rinse		PBS	Campy-Cefex
DeVillena et al. (2022)	Commercial plant before/after study	Rehang	Rehang	Chlorine/ PAA	Carcass rinse	BPW	BPW	Tempo
Bailey et al. (2019)	Commercial plant before/after study	Before IOBW	Evisceration	None	Carcass rinse	nBPW	Bolton Broth	Campy-Cefex
Berrang and Dickens (2000)	Commercial plant before/after study	Before IOBW	Evisceration	Chlorine	Carcass rinse	Distilled Water	PBS	Campy-Cefex
Cason et al. (1997)	Commercial plant before/after study	Post-Pick/ PreChill	Evisceration	None	Carcass Rinse	PBS	Campylobacter Enrichment Broth	Campy-Cefex
DeVillena et al. (2022)	Commercial plant before/after study	Before IOBW	Evisceration	Chlorine	Carcass rinse	BPW	BPW	Tempo
Bailey et al. (2019)	Commercial plant before/after study	Pre-Chill Spray	Carcass Wash	CPC/PAA	Carcass rinse	nBPW	Bolton Broth	Campy-Cefex
Bashor et al. (2004)	Commercial plant before/after study	IOBW	Carcass Wash	None/TSP/ ASC	Carcass rinse	Phosphate BPW		CCDA
Berghaus et al. (2013)	Commercial plant before/after study	IOBW	Carcass Wash	None	Carcass rinse	BPW	Bolton Broth	Campy-Cefex
Berrang and Dickens (2000)	Commercial plant before/after study	IOBW	Carcass Wash	Chlorine	Carcass rinse	Distilled Water	PBS	Campy-Cefex
DeVillena et al. (2022)	Commercial plant before/after study	IOBW	Carcass Wash	Chlorine/ PAA	Carcass rinse	BPW	BPW	Tempo
Kemp et al. (2001)	Commercial plant before/after study	Pre-Chill Spray	Carcass Wash	None/ASC	Carcass rinse	Butterfield's Buffer	Hunt Broth	Campy-Line/ mCCDA
Oyarzabal et al. (2004)	Commercial plant before/after study	IOBW	Carcass Wash	None	Carcass rinse	BPW	Hunt Broth	Campy-Cefex
Thames et al. (2022)	Commercial plant before/after study	Pre-Chill Spray	Carcass Wash	PAA	Carcass rinse	BPW	Bolton Broth	Campy-Cefex
Zhang et al. (2011)	Commercial plant before/after study	Pre-Chill Spray	Carcass Wash	CPC	Carcass rinse	BPW	Bolton Broth	Campy-Cefex
Bailey et al. (2019)	Commercial plant before/after study	Pre-Chill Immersion	Carcass Wash	PAA	Carcass rinse	nBPW	Bolton Broth	Campy-Cefex
Bailey et al. (2019)	Commercial plant before/after study	Immersion Chill	Carcass Chill	PAA	Carcass rinse	nBPW	Bolton Broth	Campy-Cefex
Bashor et al. (2004)	Commercial plant before/after study	Immersion Chill	Carcass Chill	None	Carcass rinse	Phosphate BPW		CCDA
Bauermeister et al. (2008)	Commercial plant before/after study	Post-Chill Immersion	Carcass Chill	Chlorine/ PAA	Carcass rinse	BPW	Bolton Broth	mCCDA
Berghaus et al. (2013)	Commercial plant before/after study	Immersion Chill	Carcass Chill	Chlorine	Carcass rinse	BPW	Bolton Broth	Campy-Cefex
Berrang et al. (2007)	Commercial plant before/after study	Immersion Chill	Carcass Chill	Chlorine	Carcass rinse		PBS	Campy-Cefex
Berrang, Dickens, et al. (2000)	Commercial plant before/after study	Immersion Chill	Carcass Chill	Chlorine	Carcass rinse	Distilled Water	PBS	Campy-Cefex
Cason et al. (1997)	Commercial plant before/after study	Immersion Chill	Carcass Chill	None	Carcass rinse	PBS	PBS	Campy-Cefex
Demirok et al. (2013)	Commercial plant before/after study	Immersion Chill	Carcass Chill	None/Chlorine	Carcass rinse	BPW	Bolton Broth	Campy-Cefex
DeVillena et al. (2022)	Commercial plant before/after study	Post-Chill Immersion	Carcass Chill	Chlorine/PAA	Carcass rinse	BPW		Tempo
Northcutt et al. (2003)	Pilot plant before/after study	Immersion Chill	Carcass Chill	Chlorine	Carcass rinse	PBS	PBS	Campy-Cefex
Oyarzabal et al. (2004)	Commercial plant before/after study	Immersion Chill	Carcass Chill	None/ASC	Carcass rinse	BPW	Hunt Broth	Campy-Cefex/Campy-Line/Karmali/mCCDA
Son et al. (2007)	Commercial plant before/after study	Immersion Chill	Carcass Chill	None	Carcass rinse	Sterile Water	Bolton Broth	CVA
Stern et al. (2001)	Commercial plant before/after study	Immersion Chill	Carcass Chill	Chlorine	Carcass rinse		CEB	Campy-Cefex
Thames et al. (2022)	Commercial plant before/after study	Post-Chill Immersion	Carcass Chill	PAA	Carcass rinse	BPW	Bolton Broth	Campy-Cefex
Zhang et al. (2011)	Commercial plant before/after study	Immersion Chill	Carcass Chill	None/Chlorine	Carcass rinse	BPW	Bolton Broth	Campy-Cefex
DeVillena et al. (2022)	Commercial plant before/after study	Immersion Tank	Parts	Chlorine/PAA	Wing rinse	BPW		Tempo
Thames et al. (2022)	Commercial plant before/after study	Immersion Tank	Parts	None/PAA	Drumstick rinse	BPW	Bolton Broth	Campy-Cefex
USDA-FSIS (2016)	Commercial Plant Performance Standard samples	NA	Ground	NA	Comminuted	BPW		
USDA-FSIS (2016)	Commercial Plant Performance Standard Samples	NA	Parts	NA	legs/breast/ wings	BPW		
(USDA-FSIS, 2016)	Commercial Plant Performance Standard Samples	NA	Ground	NA	MSC	BPW		
(USDA-FSIS, 2023)	Commercial Plant Performance Standard Samples	NA	Ground	NA	Comminuted	nBPW		
(USDA-FSIS, 2023)	Commercial Plant Performance Standard Samples	NA	Parts	NA	legs/breast/wings	nBPW		
(USDA-FSIS, 2023)	Commercial Plant Performance Standard Samples	NA	Ground	NA	MSC	nBPW		

11 studies reported intervention trials other than the control group to control *Campylobacter* prevalence. The studies reported the use of 7 different treatment types against *Campylobacter* prevalence. The interventions identified from literature and included for meta-analysis were acidified sodium chlorite (ASC, *n* = 3), air chill (AC, *n* = 2), cloacal plug (CP, *n* = 2), cetylpyridinium chloride (CPC, *n* = 2), high scalding pH (*n* = 1), peroxyacetic acid (PAA *n* = 4), and trisodium phosphate (TSP *n* = 1). Chemical applications were applied either through an immersion application in a dip tank (*n* = 18) or a spray application (*n* = 4).

**Fig. 3:**
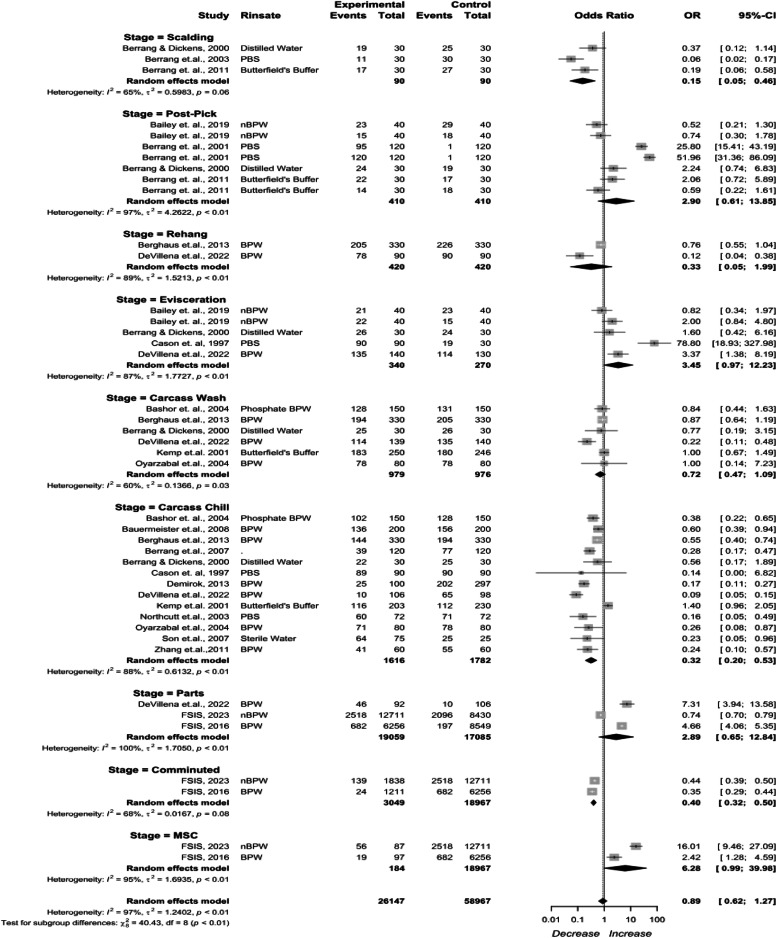
*Campylobacter* prevalence change per stage without reported interventions or chlorine. The random effects model results represent the prevalence change per stage for the control group that includes studies without reported interventions or chlorine. Results > 1 indicate an increase in concentration. Results < 1 indicate a decrease. Results = 1 indicate no change. Abbreviations: BPW = buffered peptone water, nBPW = neutralizing buffered peptone water, PBS = phosphate buffered saline.

***Meta-analysis for Campylobacter Prevalence Changes per Processing Stage for Control Group.*** Receiving (incoming prevalence) was estimated at 84 % (95 % CI: 61 %–95 %). Prevalence change, calculated as odds ratio (OR), was obtained for 9 stages (Fig. 3). Scalding being 0.15 (95 % CI: 0.05 to 0.46), Feather Pick being 2.90 (95 % CI: 0.61 to 13.85), Rehang being 0.33 (95 % CI: 0.05 to 1.99), Evisceration being 3.45 (95 % CI: 0.97 to 12.23), Carcass Wash (IOBW) being 0.72 (95 % CI: 0.47 to 1.09), Carcass Chill (Immersion Chiller) being 0.32 (95 % CI: 0.20 to 0.53), Cut-Up Parts being 2.89 (95 % CI: 0.65 to 12.84). Comminuted poultry and MSC were calculated using only the FSIS performance standard samples with an OR of 0.40 (95 % CI: 0.32 to 0.50) and 6.28 (95 % CI: 0.99 to 39.98) respectively. Data to obtain prevalence for parts and comminuted poultry from commercial processing facilities was limited. USDA-FSIS datasets of 2016 and 2023 were included as additional evidence of parts and comminuted sampling to add additional data points.

Scalding represents the highest prevalence reduction. Post-pick represents the highest increase in *Campylobacter* prevalence. Additional reductions are represented in carcass wash, and carcass chilling. Subsequent processes for cut up represent an increase while comminuted products represent a decrease, and MSC an increase. High heterogeneity existed for the stage (*I^2^* = 97 %, *p* < 0.01), indicating inconsistency in the dataset. Due to the substantially high heterogeneity across trials, no definitive conclusions can be made about the effectiveness of each processing stage.

**Fig. 4:**
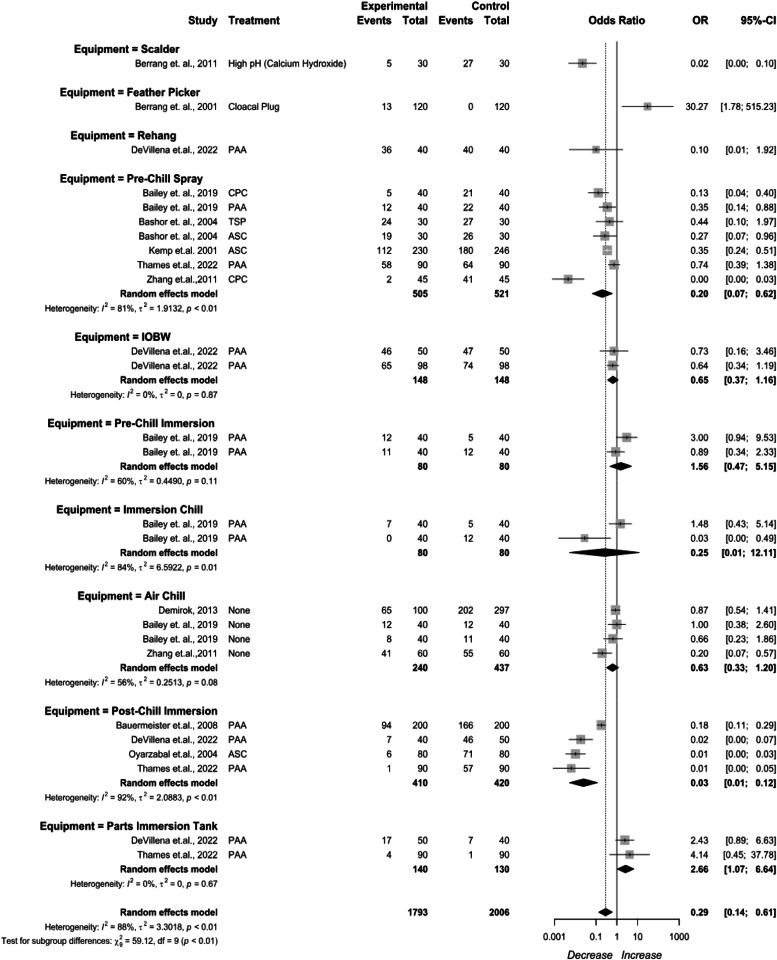
*Campylobacter* prevalence change from intervention application method. The random effects model results represent the prevalence change of the equipment used to apply interventions. Results > 1 indicate an increase in concentration. Results < 1 indicate a decrease. Results = 1 indicate no change. Abbreviations: ASC = acidified sodium chlorite, CPC = cetylpyridinium chloride, PAA = peroxyacetic acid, TSP = trisodium phosphate.

***Meta-analysis for Interventions Against Campylobacter.*** Several pre-chill and post-chill interventions were grouped by application methods and compared *Campylobacter* prevalence change (Fig. 4). Pre-chill interventions against *Campylobacter* included scalding, feather picking, rehang, inside-outside bird washers (IOBW), pre-chill sprays and immersion tanks. A treatment to increase pH was analyzed for the scalding stage being 0.02 (95 % CI: 0.00 to 0.10). A cloacal plug (CP) used during feather picking being 30.27 (95 % CI: 1.78 to 515.23). PAA at the rehang stage being 0.10 (95 % CI:0.01 to 1.92), Pre-chill spray being 0.20 (95 % CI: 0.07 to 0.02), IOBW being 0.65 (95 % CI: 0.37 to 1.16). Pre-chill immersion being 1.56 (95 % CI: 0.47 to 5.15).

Immersion and air chill as an overall application method were included. Immersion chill being 0.25 (95 % CI: 0.01 to 12.11) and air chill being 0.63 (95 % CI:0.33 to 1.20). Post-chill interventions against *Campylobacter* included post-chill immersion tanks for whole birds, and immersion tanks for cut up parts. The post-chill immersion tanks for whole birds being 0.03 (95 % CI: 0.01 to 0.12), and immersion tanks for cut up parts being 2.66 (95 % CI: 1.07 to 6.64). High heterogeneity existed for the stage (I^2^ = 86 %, *p* < 0.01), indicating inconsistency in the dataset.

**Fig. 5:**
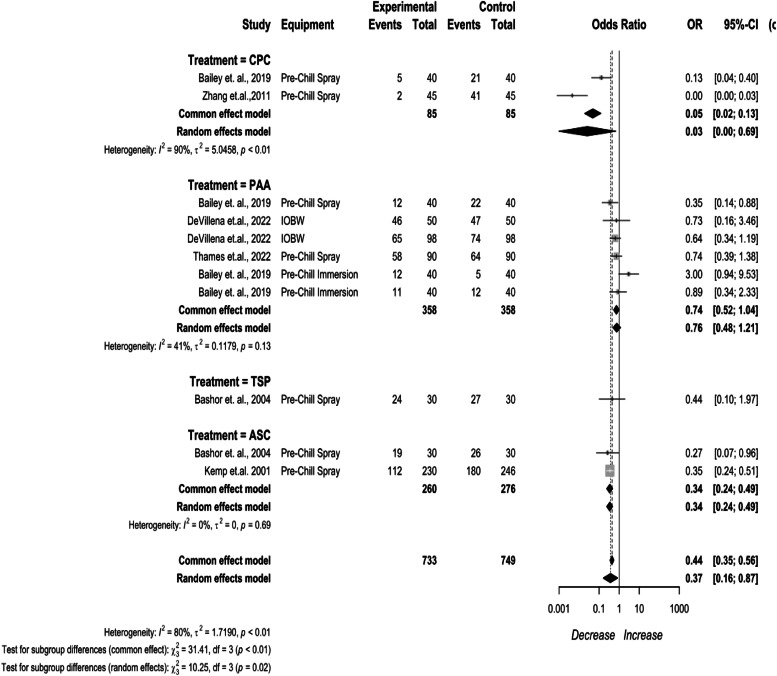
*Campylobacter* prevalence change for pre-chill interventions. The random effects model results represent the prevalence change of the interventions applied prior to the chilling process. Results > 1 indicate an increase in concentration. Results < 1 indicate a decrease. Results = 1 indicate no change. Abbreviations: ASC = acidified sodium chlorite, CPC = cetylpyridinium chloride, IOBW = inside-outside bird washers, PAA = peroxyacetic acid, TSP = trisodium phosphate.

The reported pre-chill chemical interventions included CPC, PAA, TSP, and ASC (Fig. 5). CPC being 0.03 (95 % CI: 0.00 to 0.69), PAA being (95 % CI: 0.48 to 1.21), TSP being 0.44 (95 % CI: 0.10 to 1.97), and ASC being 0.34 (95 % CI: 0.24 to 0.49). High heterogeneity existed for the stage (I^2^ = 80 %, *p* < 0.01), indicating inconsistency in the dataset.

**Fig. 6:**
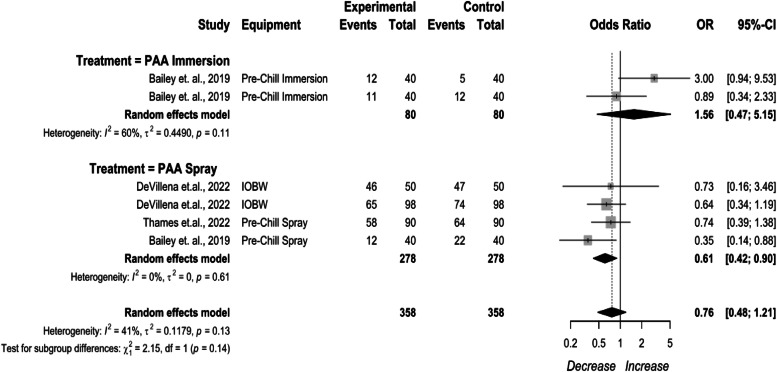
Pre-chill Peroxyacetic Acid (PAA) *Campylobacter* prevalence change. The random effects model results represent the prevalence change of PAA applied prior to the chilling process. Results > 1 indicate an increase in concentration. Results < 1 indicate a decrease. Results = 1 indicate no change. Abbreviations: IOBW = inside-outside bird washers, PAA = peroxyacetic acid.

Pre-chill PAA treatments were compared by application method (Fig. 6). PAA as a pre-chill immersion treatment being 1.56 (95 % CI: 0.47to 5.15), and as a pre-chill spray being 0.61 (95 %CI: 0.42 to 0.90). The included study for the PAA as a pre-chill immersion treatment included a manual rehanging stage plus a dip treatment prior to entering an air chiller. Possible cross contamination and the sampling procedure did not adequately represent an automated process that is common in a commercial processing plant. It was decided to keep the treatment in the SR-MA for reference for use in future SR-MA. Typically, PAA applied through immersion results in a prevalence decrease (Leone et al., 2024).

**Fig. 7:**
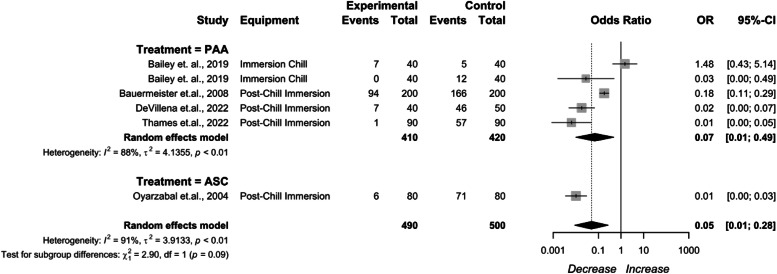
Post-chill interventions *Campylobacter* prevalence change. The random effects model results represent the prevalence change of chilling and post chill interventions. Results > 1 indicate an increase in concentration. Results < 1 indicate a decrease. Results = 1 indicate no change. Abbreviations: ASC = acidified sodium chlorite, PAA = peroxyacetic acid.

The reported post-chill immersion interventions included PAA and ASC (Fig. 7). PAA being0.07 (95 % CI: 0.01 to 0.49), and ASC being 0.01 (95 % CI: 0.00 to 0.03). High heterogeneity existed for the stage (I^2^ = 92 %, *p* < 0.01), indicating inconsistency in the dataset.

**Fig. 8:**
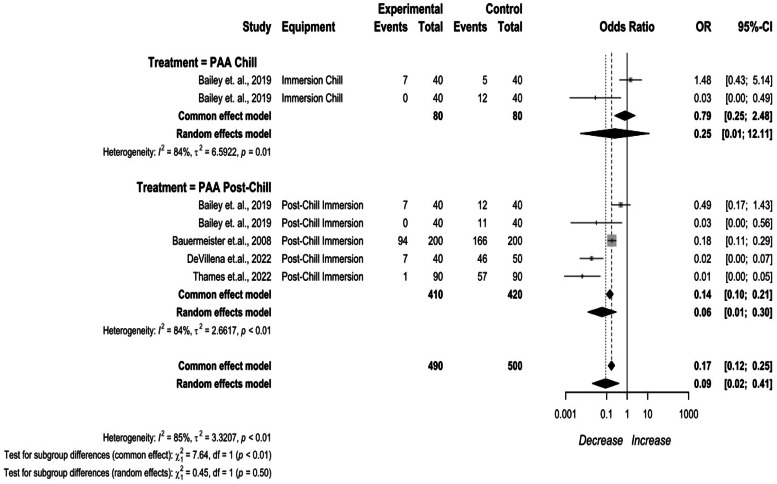
PAA Post-chill treatments prevalence change. The random effects model results represent the prevalence change of PAA applied during and after the chilling process. Results > 1 indicate an increase in concentration. Results < 1 indicate a decrease. Results = 1 indicate no change. Abbreviations: PAA = peroxyacetic acid.

Post-chill PAA treatments were compared by application method (Fig. 8). PAA applied in the immersion chiller being 0.25 (95 % CI: 0.01 to 12.11) and 0.03 (95 % CI: 0.00 to 0.24). There was high heterogeneity between studies (I^2^ = 88 %, *p* < 0.01) .

### Exposure assessment

***Baseline Model and Model Validation.*** The baseline model is defined as a basic commercial chicken processing plant in the U.S. including scalding, feather picking, rehang, evisceration, carcass washing through IOBW, immersion chilling, parts cut-up and comminuted with no reported interventions or reported chlorine use from the SR-MA. Comminuted products include ground, shredded, and minced chicken. Grinding equipment is not available in most chicken processing plants, but comminuted product is available in several further processed products (NCC, 2023). Ground products, such as mechanically separated chicken (MSC) often go to further processes that include a lethality step. Therefore, comminuted products are seldom included in raw ready-to-cook pathogen analysis. The input parameters for the baseline processing model are described in (Table 2).

**Table 2:** Input parameters for baseline model simulation from the SR-MA.

Processing Stage	Concentration Change Distribution	Unit
Receiving (Initial Prevalence)	PertAlt(5 %,0.61,0.84,95 %,0.95)	%
Scalding	PertAlt(5 %,0.05,0.15,95 %,0.46)	OR
Feather Picking	PertAlt(5 %,0.61,2.90,95 %,13.85)	OR
Rehang	PertAlt(5 %,0.05,0.33,95 %,1.99)	OR
Evisceration	PertAlt(5 %,0.97,3.45,95 %,12.23)	OR
Carcass Wash	PertAlt(5 %,0.47,0.72,95 %,1.09)	OR
Carcass Chill	PertAlt(5 %,0.20,0.32,95 %,0.53)	OR
Cut Up Parts	PertAlt(5 %,0.65,2.89,95 %,12.84)	OR
Comminuted	PertAlt(5 %,0.32,0.40,95 %,0.50)	OR

**Fig. 9:**
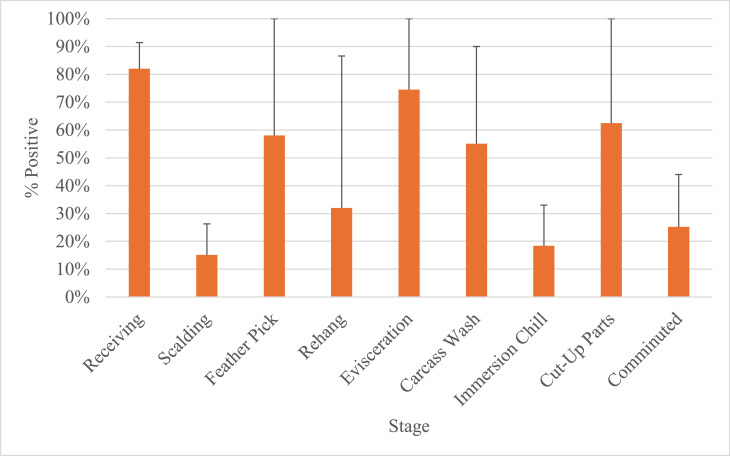
Baseline *Campylobacter* bio-map. The chart represents a simulation model of *Campylobacter* prevalence per stage without interventions or chlorine.

The simulation estimated *Campylobacter* prevalence to be incoming at 82 % (95 % CI: 71 % to 91 %, scalding at 15.16 % (95 CI: 6.52 % to 26.27 %, feather pick at 58.02 % (95 % CI: 15.04 % to 100 %, rehang at 32 % (95 % CI: 4.32 % to 86.58 %), evisceration at 74.50 % (95 % CI: 15.26 % to 100 %), carcass wash at 55.11 % (95 % CI: 11 % to 90 %), at immersion chill 18.41 % (95 %CI: 3.49 % to 33.02 %), after cut up at, 62.49 % (95 %: 9.82 % - 100 %), and comminuted at 25.21 % (95 % CI: 3.96 % to 44.01 %) (Fig. 9). The model output suggests that a processing plant can reduce *Campylobacter* prevalence for whole birds, but subsequent cut-up and grinding stages result in an increase in prevalence if additional controls are not implemented.

**Fig. 10:**
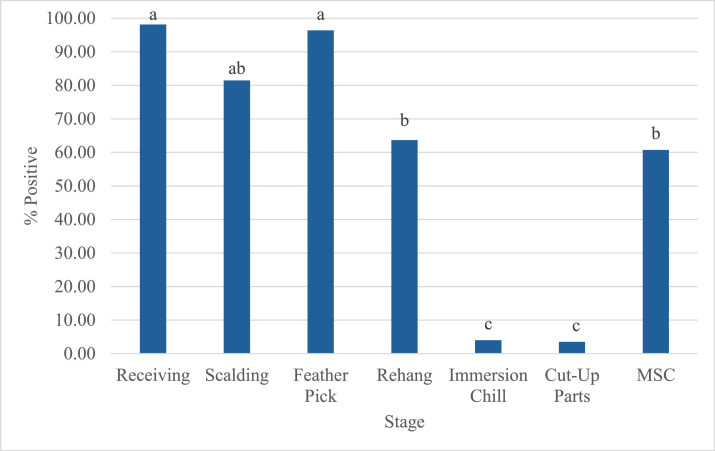
Integrator *Campylobacter* prevalence bio-map. This figure represents the pooled prevalence per stage obtained from commercial integrators. The figure includes results from the receiving, scalding, rehang, whole birds after chill, cut-up parts, and MDM stages. Samples after the post-chill stage went through a PAA application. Distinct letters on top of the stage indicate statistically significant differences (*P* < 0.05). Abbreviations: MSC = mechanically separated chicken.

Validation of the final *Campylobacter* prevalence in chicken was done by comparing the model with the *Campylobacter* prevalence estimates obtained from data from commercial processing plants in the U.S. *Campylobacter* prevalence recovered from receiving (incoming prevalence), scalding, feather pick, rehang, immersion chill, cut up parts and MSC sampling were collected from routine testing of 33 commercial processing facilities in the U.S. over the period of 2018 to 2024. (Fig. 10). The processing plants reported using PAA for chill, post-chill and cut-up processes.

The prevalence at receiving was 98.15 %, for scalding 81.48 %, feather pick 96.43 %, rehang 63.65 %, post-chill whole birds 4 %, cut up 3.5 %, and MSC 60.74 %. The decrease at rehang was significantly different when compared to receiving, scalding and feather picking (*P* < 0.05). Most of the processing plants represented in the data include a PAA or Chlorine spray treatment prior to the rehang stage. Significant reductions were represented after post-chill where whole bird samples averages a *Campylobacter* prevalence of 4 % (*P* < 0.05). Prevalence after cut up does not represent a significant change (*P* > 0.05). MSC represents a significant increase (*P* < 0.05). A sample size of 135 and the results are similar to the USDA-FSIS sampling datasets. MSC is used for further processing that undergoes a lethality step. The few samples were collected for exploration sampling. MSC is seldom sampled as raw ready-to-cook product and prevalence results cannot be an indicator of exposure to consumers.

**Fig. 11:**
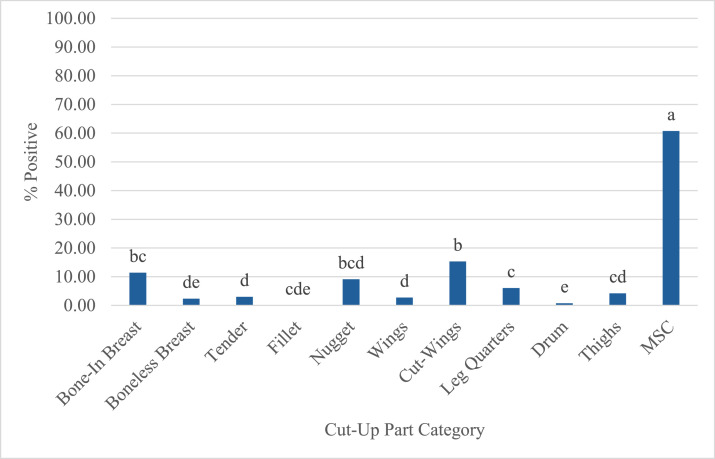
Integrator *Campylobacter* prevalence per parts categories. This figure represents the cut-up parts broken down by the categories obtained from the pooled results from commercial integrators. All cut-up parts categories were treated with PAA. Distinct letters on top of the stage indicate statistically significant differences (*P* < 0.05). Abbreviations: MSC = mechanically separated chicken, PAA = peroxyacetic acid.

Cut up parts data obtained from the commercial processing plants were organized in separate categories (Fig. 11). Results were pooled per category to compare *Campylobacter* prevalence between parts. The prevalence between parts resulted in bone-in breast 11.38 %, boneless breast 2.36 %, tender 3 %, fillet 0 %, nugget (boneless breast trim) 9.09 %, wings 2.73 %, cut-wings 15.27 %, leg quarters 6.05 %, drum 0.77 %, thighs 4.23 %, MSC 60.74 %. The cut-up parts samples were obtained after all PAA interventions were applied. Bone-in breast had a significantly higher prevalence than its boneless derivatives (boneless breast, tenders, and fillets) (*P* < 0.05). Nuggets are boneless breast trim that is used for whole breast nuggets. The prevalence of nuggets is significantly higher (*P* < 0.05) than other boneless breast products. *Campylobacter* prevalence can be influenced by additional cut-up steps. The prevalence of wings does not differ from most cut-up parts with the exception of leg quarters and bone-in parts (bone-in breast, legs). The prevalence for cutting wings is significantly different from wings and other bone-in parts except bone-in breast (*P* < 0.05). The prevalence for drums is significantly lower (*P* < 0.05) than leg quarters and thighs. There are differences in *Campylobacter* prevalence between cut-up parts. It is not clear whether handling, interventions, processing or storage conditions affect prevalence at this stage. The processing conditions at time of sampling were not provided with the data, therefore definitive conclusions of what causes the difference in prevalence distributions cannot be made from the commercial plant data.

The *Campylobacter* prevalence pattern observed in commercial plants (Fig. 10) was consistent with the baseline simulation model (Fig. 9) and SR-MA findings (Fig. 3), though absolute levels for whole birds and parts were lower due to prevalent PAA use. *Campylobacter* prevalence for comminuted products were like the prevalence of USDA-FSIS sampling results. A breakdown of comminuted categories cannot be achieved due to limited data.

***Scenario Analysis.*** 4 different single intervention application methods (air chill, immersion chill, spray, and immersion) and 4 of the most studied chemical interventions (ASC, CPC, PAA, and TSP) were selected from the SR-MA to analyze its capacity of changing a processing plant’s ability to control *Campylobacter* in whole birds, cut-up parts or comminuted chicken for a total of 13 single intervention scenarios and 1 multi-intervention scenario. The input parameters for the single intervention scenarios are described in Table 3. Table 4 includes the *Campylobacter* prevalence and efficacy estimates for all single intervention scenarios and one multi-intervention scenario for whole birds, cut-up parts and comminuted product respectively.

**Table 3:** Input distributions of processing interventions from the SR-MA used for intervention efficacy analysis.

Intervention Type	Concentration Change Distribution	Unit
IOBW-PAA	PertAlt(5 %,0.37,0.65,95 %,1.16)	OR
Pre-Chill Immersion - PAA	PertAlt(5 %,0.47,1.56,95 %,5.15)	OR
Pre-Chill Spray	PertAlt(5 %,0.07,0.20,95 %,0.62)	OR
a. CPC	PertAlt(5 %,0.00,0.03,95 %,0.69)	OR
b. PAA	PertAlt(5 %,0.42,0.61,95 %,0.90)	OR
c. TSP	PertAlt(5 %,0.10,0.44,95 %,1.97)	OR
d. ASC	PertAlt(5 %,0.24,0.34,95 %,0.49)	OR
Air Chill	PertAlt(5 %,0.33,0.63,95 %,1.20)	OR
Immersion Chill PAA	PertAlt(5 %,0.01,0.25,95 %,12.11)	OR
Post Chill Immersion - Whole Bird	PertAlt(5 %,0.01,0.03,95 %,0.12)	OR
a. PAA	PertAlt(5 %,0.01,0.07,95 %,0.49)	OR
b. ASC	PertAlt(5 %,0.00,0.01,95 %,0.03)	OR
Post Chill Immersion - Cut-Up Parts	PertAlt(5 %,1.07,2.66,95 %,6.64)	OR

**Table 4:** Intervention efficacy analysis for whole birds.

Scenario	Whole Bird Prevalence (%)	Efficacy (%)	Parts Prevalence (%)	Efficacy (%)	Comminuted Prevalence (%)	Efficacy (%)
Baseline	18.41	-	62.49	-	25.21	-
IOBW-PAA	17.14	6.90	59.27	5.15	23.91	5.16
Pre-Chill Immersion PAA	25.59	Not Effective	74.27	Not Effective	29.96	Not Effective
Pre-Chill Spray	4.58	75.12	18.96	69.66	7.66	69.62
a. CPC	2.49	86.47	10.35	83.44	4.17	83.46
b. PAA	14.52	21.13	53.23	14.82	21.48	14.80
c. TSP	11.64	36.77	42.7	31.67	17.22	31.69
d. ASC	6.42	65.13	26.63	57.39	10.75	57.36
Air Chiller	37.14	Not Effective	81.25	Not Effective	32.76	Not Effective
Immersion Chiller PAA	7.19	60.95	28.84	53.85	11.63	53.87
Post Chill Immersion Whole Birds	0.77	95.82	3.22	94.85	1.3	94.84
a. PAA	2.4	86.96	10.02	83.97	4.05	83.93
b. ASC	0.21	98.86	0.9	98.56	0.36	98.57
Post-Cut Up Immersion PAA	-	-	53.55	14.31	21.59	14.36
Incoming+ IOBW PAA+ Pre-Chill ASC Spray + Chiller PAA+ Post Chill PAA Dip + Post Cut-Up PAA Dip	2.14	88.38	6.5	89.60	2.62	89.61

PAA as an IOBW application had an efficacy of 6.90 % in whole birds, 5.15 % in cut up parts, and 5.16 % in comminuted chicken. PAA as a pre-chill immersion application was not effective at reducing prevalence in whole birds, parts, and comminuted chicken.

Pre-chill spray applications had an overall efficacy of 75.12 % in whole birds, 69.66 % in cut up parts, and 69.62 % in comminuted chicken. CPC as a pre-chill spray application had an efficacy of 86.47 % in whole birds, 83.44 % in cut up parts, 83.46 % in comminuted chicken. PAA as a pre-chill spray application had an efficacy of 21.13 % in whole birds, 14.82 % in cut up parts, and 14.80 % in comminuted chicken. TSP as a pre-chill spray had an efficacy of 36.77 % in whole birds, 31.67 % in cut up parts, and 31.69 % in comminuted chicken, ASC as a pre-chill spray had an efficacy of 65.13 % in whole birds, 57.39 % in cut up parts, and 57.36 % in comminuted chicken.

Air chill as a substitute for immersion chill was not effective at reducing prevalence. PAA as an immersion chill application had an efficacy of 60.95 % in whole birds, 53.85 % in cut up parts, and 53.87 % in comminuted chicken.

Post-chill immersion applications in whole birds had an overall efficacy of 95.82 % in whole birds, 94.85 % in cut up parts, and 94.84 % in comminuted chicken. PAA as a post-chill immersion application had an efficacy of 86.96 % in whole birds, 83.97 % in cut up parts, and 83.93 % in comminuted chicken. ASC as a post-chill immersion application had an efficacy of 98.86 % in whole birds, 98.56 % in cut up parts, and 98.57 % in comminuted chicken. PAA as a post-cut up immersion application had an efficacy of 14.31 % in cut up parts, and 14.36 % in comminuted chicken.

A multi-intervention approach including the incoming prevalence, PAA in the IOBW, pre-chill ASC spray, PAA in the immersion chiller, PAA as a post-chill immersion, and PAA as a post-cut up application had an efficacy of 88.30 % in whole birds, 89.60 % in cut up parts, and 89.61 % in comminuted chicken.

## Discussion

### Meta-analysis for *Campylobacter* baseline prevalence per processing stage

The SR-MA identified the stage-specific variation in *Campylobacter* concentration across processing. The processing stages up to immersion chilling reduces *Campylobacter* prevalence on whole bird carcasses with minimal to no interventions. This aligns with global bio-mapping studies from commercial processing plants (Betancourt-Barszcz et al., 2024; Chavez-Velado et al., 2024; DeVillena et al., 2022; Kingsbury et al., 2023; Vargas et al., 2023).

However, establishing baseline *Campylobacter* prevalence for parts and comminuted products and MSC directly from the SR-MA was precluded by a lack of before-after studies from commercial processing facilities, therefore USDA-FSIS samples for performance standards were used. Reductions are not sustained in subsequent stages without the use of interventions. Prevalence was higher for cut-up parts and MSC. The prevalence of cut-up parts and MSC obtained from the meta-analysis resulted in a significant increase in prevalence. This suggests that growth preventing conditions (e.g. time and temperature, product-contact surface sanitation) is not sufficient to prevent prevalence increases without interventions.

Estimates for *Campylobacter* prevalence on comminuted chicken suggests that prevalence levels from prior stages are sustained through a grinding process if growth preventing conditions (e.g. time and temperature) are maintained. These estimates remain unvalidated against commercial processing or with published studies, primarily because most U.S. ground poultry undergoes further processing (e.g., thermal inactivation) and is exempt from the regulatory sampling as it’s not sold as a raw ready-to-cook product.

The baseline SR-MA presented limitations. The heterogeneity and within study variation was high for both prevalence and prevalence change analysis. The study required dividing the studies into several subgroups and even though a mean value was established and was verified by overall comparisons to commercial sampling, the subgroup analysis had very few studies with sufficient data points to perform adequate within study and between study variation analysis.

The USDA-FSIS data set presented limitations. Sample totals differed between whole bird, parts, comminuted, and MSC making a prevalence change per stage comparison difficult. Establishments were difficult to identify from the dataset and within establishment comparisons were not performed. In addition, the interventions and conditions at time of sampling were not recorded. The samples cannot be distinguished based on processing conditions. The 2016 dataset was used to represent prevalence at the start of parts performance standard implementation and buffered peptone water as the rinse solution (USDA-FSIS, 2016). The 2023 dataset was used to represent samples collected with neutralizing buffered peptone water as the rinse solution (USDA-FSIS, 2023).

### Meta-analysis for interventions against *Campylobacter*

Most interventions included in the SR-MA could reduce *Campylobacter* prevalence on carcasses and on cut-up parts, but evidence is often limited to single studies, precluding robust assessment of heterogeneity. For example, only one study evaluated cloacal plugs, which limited cross-contamination during feather picking compared to controls; more research is needed to optimize such novel approaches(Berrang et al., 2018, 2001).

Heterogeneity was higher for most chemical interventions, reflecting varied study numbers and methodologies (sampling, matrices). PAA emerged as the dominant U.S. intervention, applied via immersion or spray pre- or post- carcass chiller. PAA is also widely used in carcass chillers. Therefore, several comparisons were achieved. Only one study was included as pre-chill PAA immersion and there is uncertainty that the setting reflects a typical processing plant. One study obtained from the SR-MA observed dwell times like a typical immersion chiller in processing plants and it was decided to analyze its effect by grouping the study along with studies observing post-chill intervention effect. The immersion chill study was also analyzed separate from the post-chill interventions to obtain an OR for efficacy analysis The results for PAA are consistent to past reviews (Cano et al., 2021; Oyarzabal, 2005). The decision to use PAA over other available chemical antimicrobials is due to cost-effectiveness and implementability.

Lesser-known interventions, such as cloacal plugs, cloacal washes, UV, etc. were included for comparison. A single study due to limited available literature was used, which reduces statistical power and increases the potential for bias, limiting the reliability and interpretability of the results. However, these results were still presented as a reference for the reader’s interest, highlighting potential research gaps and they require further research for optimization and potential integration as complementary controls.

### Exposure assessment

The baseline simulation model captured *Campylobacter* prevalence patterns observed in the SR-MA and the validation data, though simulated concentrations for whole birds and parts were higher than commercial validation data. This discrepancy is likely attributable to widespread PAA use in commercial processing plants, absent in the baseline model. Importantly, incorporating interventions into the model yielded concentrations matching validation data, confirming the baseline as a valid representation of a U.S. plant without interventions. This validated model provides a foundation for simulating diverse intervention scenarios.

The key model finding indicate reduced post-chill *Campylobacter* prevalence followed by an increase cut-up and a reduction after a grinding process. The model indicates that temperature control (inhibiting growth) and sanitation (minimizing cross-contamination) may be insufficient to prevent prevalence increases. While interventions like PAA significantly reduce pre-cut *Campylobacter* prevalence, they show limited additional post-cut reduction. Applying interventions at the post-chill plus the post-cut up stage helps maintain low levels and may confer carryover effects to subsequent stages like grinding or packaging. Post-processing concentrations had variations across cut-up parts type and prevalence was higher comparable to whole carcasses, suggesting that exposure risk varies by part category. Simulated comminuted product concentrations serve as a crucial benchmark due to the lack of comparable commercial data.

Simulating a chicken processing plant presented several limitations. Model development faced data scarcity, particularly for cut parts and comminuted products, due to limited commercial/ pilot plant trials and restricted access for controlled studies (e.g., testing reduced/ no interventions was precluded by regulations). Some uncertainties include the sampling locations and sampling matrices to determine incoming prevalence. Even though the incoming prevalence was comparable to incoming *Campylobacter* prevalence obtained from commercial processing plants, the SR-MA contained studies where incoming prevalence was determined by sampling at different locations before the scalder and sampled several matrices from carcass rinses to cecal content (Berghaus et al., 2013; Berrang, Buhr, et al., 2000; Kotula and Pandya, 1995; Mead et al., 1995; Potturi-Venkata et al., 2007; Stern and Robach, 2003). Another uncertainty is the impact of cross contamination on *Campylobacter* prevalence throughout the process. Cross contamination in feather picking is known to occur, but the rate of cross-contamination per bird and how much equipment contributes to cross contamination throughout subsequent steps was not factored in the simulations process perhaps underestimating *Campylobacter* prevalence in the model. The effect on the different sampling locations could not be included. A source of variability included the wide range of sample rinse volumes, detection methods, and sample matrices. Carcass rinse data extracted from the studies ranged from 200 mL to 400 mL. There are differences in the amount of *Campylobacter* concentrations obtained from different rinse volumes (Williams et al., 2010). The type of rinse and the type of enrichment and plating method have different sensitivities that may influence concentration data from studies (Gonsalves et al., 2016; Hiett, 2017; Line et al., 2001). The SR-MA was sufficient in providing data for the simulation models.

### Intervention efficacy

The intervention efficacy analysis confirmed that single interventions can significantly reduce *Campylobacter* prevalence. Air chilling was included in the scenario analysis as an alternative chilling stage. Air chilling was ineffective for *Campylobacter* control, necessitating validation of complementary pre-/ post-chill interventions. Post-chill immersion interventions were the most effective. Pre-chill interventions are less effective than post-chill interventions. *Campylobacter* is present at higher levels and chicken carcasses may not be exposed to the intervention for long periods of time prior to chilling. Intestinal content, debris, or tissues that contain high levels of *Campylobacter* may still be present at pre-chill stages. Post-chill interventions are applied after all mayor *Campylobacter* contamination sources like feathers, viscera, and intestinal content have been removed and the carcasses have gone through a washing process to remove all visible debris. This allows the chemical to act on the actual product and not compete with other organic material that can lower its effectiveness. There may be a carryover factor in these results that may overestimate the results. Chemical interventions, like PAA, are the last hurdle that is applied in a commercial processing setting before packaging or grinding.

The multiple intervention scenarios analyzing the application of PAA resulted in prevalence comparable to the *Campylobacter* performance standard limits. It is important to note that single interventions are effective, but a multi-hurdle system, including pre-chill and post-chill interventions is most effective against *Campylobacter*.

The single intervention and multiple interventions model present *Campylobacter* prevalence after chemical interventions comparable to the commercial processing bio-map. These results validate that the baseline model without interventions presents a possible outcome of *Campylobacter* prevalence if interventions are not applied and it can be used as a starting point to model future exposure assessments modules. However, the model presents several limitations. Much of the data utilized for intervention efficacy analysis was extracted from a limited number of studies. PAA is the most used chemical antimicrobial in processing thus more studies are available, particularly for pre- and post-chill applications. Models studying the variations in immersion chilling will allow for better assessments. The limited number of studies limits the amount of data points used to reduce variation in the results. The general risk assessment model only analyzed overall intervention data. It did not consider different concentrations of the chemical, contact time and pH levels, which can influence the effectiveness of many of these interventions. Other lesser-known interventions from the SR-MA were not included because of the limited number of studies available, and commonly used chemicals were only considered for this analysis.

In conclusion, the SR-MA enabled construction of a validated baseline model simulating *Campylobacter* prevalence in U.S. processing plants *without* interventions. Key implications:

• **Process Control:** Significant reductions occur naturally during immersion chilling and are not maintained through cutting/grinding via temperature control and sanitation.
• **Intervention Strategy:** Post-chill chemical applications are optimal. Multi-hurdle approaches minimize cross-contamination and reduce part-to-part variability, yielding uniform prevalence (often <10 %) across all products.
• **Uniform Exposure Risk:** Finished products (whole carcasses, parts, comminuted) pose comparable *Campylobacter* exposure risk due to concentration equalization during processing.
• **Critical Data Gap:** Cut parts dominate U.S. consumption yet remain underrepresented in risk assessments. Comminuted products lack commercial validation data.

While multi-hurdle interventions lower risk, they cannot eliminate *Campylobacter*. Future work must focus on a) Validating interventions for air-chilled products, b) Expanding cut-part and comminuted product sampling, c) Assessing novel chemical/non-chemical interventions and d) Evaluating pre- and post-processing mitigation steps.

## CRediT authorship contribution statement

**Rafael E. Rivera:** Writing – review & editing, Writing – original draft, Visualization, Validation, Supervision, Software, Resources, Project administration, Methodology, Investigation, Funding acquisition, Formal analysis, Data curation, Conceptualization. **Jinquan Wang:** Writing – review & editing, Writing – original draft, Visualization, Validation, Software, Methodology, Formal analysis, Conceptualization. **Abhinav Mishra:** Writing – review & editing, Supervision, Software, Methodology, Formal analysis. **Harshavardhan Thippareddi:** Writing – review & editing, Validation, Supervision, Conceptualization. **Sanjay Kumar:** Writing – review & editing. **Manpreet Singh:** Writing – review & editing, Supervision, Project administration.

## Disclosures

The authors declare the following financial interests/personal relationships which may be considered as potential competing interests:

Rafael E Rivera Betancourt reports administrative support was provided by US Poultry and Egg Association. If there are other authors, they declare that they have no known competing financial interests or personal relationships that could have appeared to influence the work reported in this paper.
